# A spasticity model based on feedback from muscle force explains muscle activity during passive stretches and gait in children with cerebral palsy

**DOI:** 10.1371/journal.pone.0208811

**Published:** 2018-12-07

**Authors:** Antoine Falisse, Lynn Bar-On, Kaat Desloovere, Ilse Jonkers, Friedl De Groote

**Affiliations:** 1 Department of Movement Sciences, KU Leuven, Leuven, Belgium; 2 Department of Rehabilitation Sciences, KU Leuven, Leuven, Belgium; 3 Amsterdam UMC, Vrije Universiteit Amsterdam, Department of Rehabilitation Medicine, Amsterdam Movement Sciences, Amsterdam, Netherlands; University of L'Aquila, ITALY

## Abstract

Muscle spasticity is characterized by exaggerated stretch reflexes and affects about 85% of the children with cerebral palsy. However, the mechanisms underlying spasticity and its influence on gait are not well understood. Here, we first aimed to model the response of spastic hamstrings and gastrocnemii in children with cerebral palsy to fast passive stretches. Then, we evaluated how the model applied to gait. We developed three models based on exaggerated proprioceptive feedback. The first model relied on feedback from muscle fiber length and velocity (velocity-related model), the second model relied on feedback from muscle fiber length, velocity, and acceleration (acceleration-related model), and the third model relied on feedback from muscle force and its first time derivative (force-related model). The force-related model better reproduced measured hamstrings and gastrocnemii activity during fast passive stretches (coefficients of determination (R^2^): 0.73 ± 0.10 and 0.60 ± 0.13, respectively, and root mean square errors (RMSE): 0.034 ± 0.031 and 0.009 ± 0.007, respectively) than the velocity-related model (R^2^: 0.46 ± 0.15 and 0.07 ± 0.13, and RMSE: 0.053 ± 0.051 and 0.015 ± 0.009), and the acceleration-related model (R^2^: 0.47 ± 0.15 and 0.09 ± 0.14, and RMSE: 0.052 ± 0.050 and 0.015 ± 0.008). Additionally, the force-related model predicted hamstrings and gastrocnemii activity that better correlated with measured activity during gait (cross correlations: 0.82 ± 0.09 and 0.85 ± 0.06, respectively) than the activity predicted by the velocity-related model (cross correlations: 0.49 ± 0.17 and 0.71 ± 0.22) and the acceleration-related model (cross correlations: 0.51 ± 0.16 and 0.67 ± 0.20). Our results therefore suggest that force encoding in muscle spindles in combination with altered feedback gains and thresholds underlie activity of spastic muscles during passive stretches and gait. Our model of spasticity opens new perspectives for studying movement impairments due to spasticity through simulation.

## Introduction

Cerebral palsy (CP) is the most common cause of physical disability in children. It is described as a group of permanent disorders that are attributed to lesions occurring in the developing brain. These disorders are accompanied by secondary musculoskeletal problems including muscle spasticity that affects about 85% of the children with CP [1,2]. Spasticity is commonly defined by a velocity-dependent increase in tonic stretch reflexes resulting from hyper-excitability of the stretch reflex [3]. The most common clinical diagnoses of spasticity are exaggerated tendon tap reflexes and hypertonia that induce a velocity-dependent resistance of spastic muscles to stretch [4]. Yet, despite the vast amount of research, the mechanisms underlying spasticity and the influence of spasticity on functional motions such as gait are not well understood. In this study, we use a simulation-based approach to model the spastic response to muscle stretch and we evaluate how this model applies to gait.

Spasticity is qualitatively assessed by passively moving a joint while grading the resistance, typically using the Modified Ashworth Scale (MAS) [5] or the Modified Tardieu Scale (MTS) [6]. To further quantify the level of spastic involvement, Bar-On et al. [7] developed instrumented tests that yield better accuracy and reliability than the MAS and MTS. During these tests, later referred to as instrumented passive spasticity assessments (IPSAs), passive muscles are stretched at different velocities while collecting biomechanical (joint moments, angular positions and velocities) and electrophysiological (electromyography (EMG)) data that quantify the resistance to the imposed motion. These tests therefore provide valuable information to comprehensively describe spasticity.

Spasticity is believed to emerge from exaggerated reflex activity in response to muscle spindle firing. In unaffected muscles, fast conducting group Ia nerve fibers mediate the activity of short-latency reflexes from the spindles to the spinal cord [4]. The central motor lesions accompanying CP are thought to affect the strength and sensitivity of this reflex loop [4,8], inducing exaggerated reflexes upon muscle stretch, i.e. spasticity.

Models of spasticity explain the increase in muscle activity following lengthening through exaggerated proprioceptive feedback from the spindles. Since it is widely thought that the spindle proprioceptive receptors encode information about muscle length and velocity changes [9,10], spasticity has been modeled based on muscle length and velocity feedback [11–14]. In particular, van der Krogt et al. [14] proposed a spasticity model based on velocity feedback. This model captured the salient features of the spastic response observed during fast passive stretches of the hamstrings in CP children but did not reproduce the oscillations in the measured muscle activity (EMG), that were not reflected in fiber velocity, and the sustained muscle activity following the stretch.

Recent findings suggest that muscle spindle firing might be more directly related to muscle force than to muscle length and velocity. In particular, Blum et al. [15] showed that history-dependent transients of spindle firing are not uniquely related to muscle fiber lengths and velocities. Further, they demonstrated that muscle spindle firing can be explained based on encoding of fiber force and its first time derivative across a wide range of stretch conditions, whereas length- and velocity-related variables cannot explain spindle firing during history-dependent conditions such as short-range stiffness. Their results therefore suggest that spindle proprioceptive receptors encode information about muscle force rather than length. This is in accordance with existing spindle models that describe spindle firing based on the stretch in a spring element representing the sensory zone, which is proportional to the intrafusal muscle fiber force [16–18], and with older *in vitro* studies relating peaks in muscle spindle firing rates to muscle force transients at stretch onset [19,20]. Since spasticity is believed to result from an exaggerated response to spindle firing, a model of spasticity based on feedback from muscle force and its first time derivative should be considered. To the authors’ knowledge, such model of spasticity has never been proposed.

While spasticity manifests during passive muscle stretches, its influence during functional motions such as gait remains subject to debate. In their review, Dietz and Sinkjaer [4] reported that the clinical signs of spasticity, i.e. exaggerated tendon tap reflexes and muscle hypertonia, are little related to the functional spastic motion disorders. They further argued that exaggerated reflexes have a minor role in spastic motion disorders whereas secondary changes in mechanical muscle fiber properties have a major role. These conclusions are in line with several studies that reported a lack of correlation between spasticity as diagnosed during passive motions and determinants of gait. Ada et al. [21] showed that tonic stretch reflexes in the gastrocnemii, provoked in conditions that simulated gait, were of similar magnitude in stroke patients and controls. This suggested no influence of spasticity on gait whereas most stroke patients exhibited exaggerated resting tonic stretch reflexes indicating spasticity in a relaxed state. Marsden et al. [22] reported no correlation between the degree of knee flexion during walking and spasticity of the knee extensors as assessed during passive stretches in people with hereditary and sporadic spastic paraparesis. Willerslev et al. [23] showed that the contribution of sensory feedback to soleus activity during swing was not larger in CP children than in typically developing children and that soleus activity was observed to the same extent during swing in both groups. They therefore concluded that spasticity was unlikely to contribute to altered ankle kinematics. In contrast, other studies could relate measures of spasticity to determinants of gait suggesting that spasticity affects walking. Damiano et al. [24] showed that CP children who exhibited increased stretch reflexes in the hamstrings and quadriceps during passive stretches had lower knee angular velocities during the swing phase of gait. Tuzson et al. [25] reported similar results as well as a correlation between the spastic threshold velocity, expressed in terms of knee angular velocity, and the peak knee angular velocity during fast walking in CP children. Further, spasticity is often assumed to affect walking performance. For example, it is thought that rectus femoris spasticity leads to abnormal activity during early swing, limiting knee flexion during that phase [26,27]. In summary, the relation between spasticity measures and gait as well as the influence of spasticity on walking performance are still under debate.

Musculoskeletal modeling and simulations have been used to investigate whether spasticity, as assessed during passive motions, e.g. with IPSA, affects gait. These tools can provide information about the muscle-tendon units, e.g. muscle fiber length, velocity, force, that might contribute to our understanding of the mechanisms underlying spasticity. Bar-On et al. [28] have used modeling to relate muscle-tendon lengthening velocity during passive stretches of the gastrocnemii and hamstrings of CP children and muscle-tendon velocities during the swing phase of gait. They reported that the peak muscle-tendon velocity during swing exceeded the stretch reflex thresholds, i.e. the muscle-tendon velocity at reflex onset, for the hamstrings but not for the gastrocnemii. Additionally, they could relate stretch reflex thresholds with peak muscle-tendon velocity during gait for both muscle groups at specific walking speeds. Finally, they reported no correlation between EMG measured during passive stretches and peak muscle-tendon lengthening velocity during gait. That study however only compared thresholds expressed in terms of muscle-tendon lengthening velocity and did not evaluate other muscle variables that have been related to muscle spindle firing such as muscle fiber length, velocity or force. Additionally, there was no attempt to model the spastic response to passive stretches and to evaluate how that model would apply to gait.

The contribution of this study is twofold. First, we developed three spasticity models and compared their ability to explain measured reflex muscle activity during passive muscle stretches in CP children. All three models described reflexes through proprioceptive feedback. The velocity-related model relied on muscle fiber length and velocity feedback. The acceleration-related model relied on muscle fiber length, velocity, and acceleration feedback. The force-related model relied on feedback from muscle force and its first time derivative (*dF*/*dt*). We hypothesized, based on the aforementioned findings from Blum et al. [15], that the force-related model would better reproduce the measured muscle activity than the velocity- and acceleration-related models. Second, we evaluated how well muscle activity predicted by the three models correlated with measured muscle activity from CP children during gait. We found that the force-related model could explain muscle activity during passive stretches and gait whereas the velocity- and acceleration-related models could not.

## Materials and methods

### Experimental data

We collected experimental data from six CP children (four with unilateral and two with bilateral spastic involvement) (Table 1). As part of their treatment, three children (two with unilateral and one with bilateral spastic involvement) received a botulinum toxin-A (BTX) injection to reduce spasticity and we collected data before and 6 to 14 weeks after treatment. We considered that the spasticity models should be able to describe different levels of spastic involvement and therefore should be valid pre- and post-BTX injection. We analyzed two muscle groups: hamstrings and gastrocnemii. In all cases, the MAS score of the hamstrings and gastrocnemii was between 1 and 2 indicating mild hyper-resistance [5]. The data were collected as part of another study and the ethical committee of the UZ / KU Leuven (Belgium) approved the protocol (s060799). All children older than 11 years and all parents signed an informed consent form.

**Table 1:** Demographic information.

	Hamstrings (n = 8)	Gastrocnemii (n = 12)
**Gender (male/female)**	3/2	3/3
**Age in years (mean ± std)**	12.4 ± 2.6	11.7 ± 2.9
**Involvement (unilateral/bilateral)**	4/1	4/2
**BTX-treatment (unilateral/bilateral)**	2/0	2/1
**Modified Ashworth Scale score (1:5)**	1: 1	1+: 5
	1+: 4	2: 7
	2: 3	
**Modified Tardieu Scale: angle in degrees (mean ± std)**	-81.4 ± 3.8	-9.2 ± 9.8

First, the children’s gait was assessed using 3D motion analysis. Each child was instrumented with retro-reflexive markers, corresponding to the lower limb plug-in-gait marker set, whose 3D locations were recorded (100 Hz) using an 8- to 15-camera motion capture system (Vicon, Oxford, UK) during walking trials. Data from 3 to 10 walking trials with valid force plate contact were selected per subject. Ground reaction forces (GRFs) (1000 Hz) and surface EMG (2000 Hz) were recorded using force plates (AMTI, Watertown, USA) and a telemetric Zerowire system (Cometa, Milan, Italy), respectively. GRFs were low-pass filtered (6 Hz) with a second-order dual-pass Butterworth filter. EMG was collected from eight muscles: rectus femoris, vastus lateralis, biceps femoris short head, semitendinosus, tibialis anterior, gastrocnemius lateralis, soleus, and gluteus medius. EMG was processed by band-pass filtering (20–400 Hz), full-wave rectification, and low pass filtering (10 Hz) using a second-order dual pass Butterworth filter.

On the same day as the gait analysis, spasticity of the medial hamstrings and gastrocnemii was assessed using IPSA (described in detail by Bar-On et al. [7]). During IPSA, hamstrings and gastrocnemii were stretched by moving knee and ankle, respectively, one at a time from a predefined position throughout the full range of motion. The stretches were performed at slow (about 15 and 10 degrees/s for testing the hamstrings and gastrocnemii, respectively), medium (about 75 and 55 degrees/s, respectively) and fast (about 200 and 100 degrees/s, respectively) velocities. Surface EMG was collected from the semitendinosus and gastrocnemius lateralis as well as from their antagonists (rectus femoris and tibialis anterior, respectively) using the same system as used for gait analysis. The electrodes remained in place between both analyses. EMG processing was comparable to Bar-On et al. [7], namely high-pass filtering (20 Hz), stop-pass filtering (49.5–50.5 Hz) to remove the observed line frequency interference, full-wave rectification, and low pass filtering (10 Hz) using a sixth-order dual pass Butterworth filter. Due to poor EMG quality, data from the hamstrings were not included for the child with bilateral involvement who received BTX treatment. This resulted in data from 20 individual muscle groups (8 hamstrings and 12 gastrocnemii) included for further analysis. The motion of the distal limb segment with respect to the proximal fixed segment was tracked using two inertial measurement units (Analog Devices, ADIS16354). The forces applied to the segment were measured using a hand-held six degrees of freedom (DOFs) load-cell (ATI Industrial Motion, mini45). The position of the load-cell relative to the joint axis was manually measured by the examiner.

### Musculoskeletal modeling and data processing

**Fig 1:**
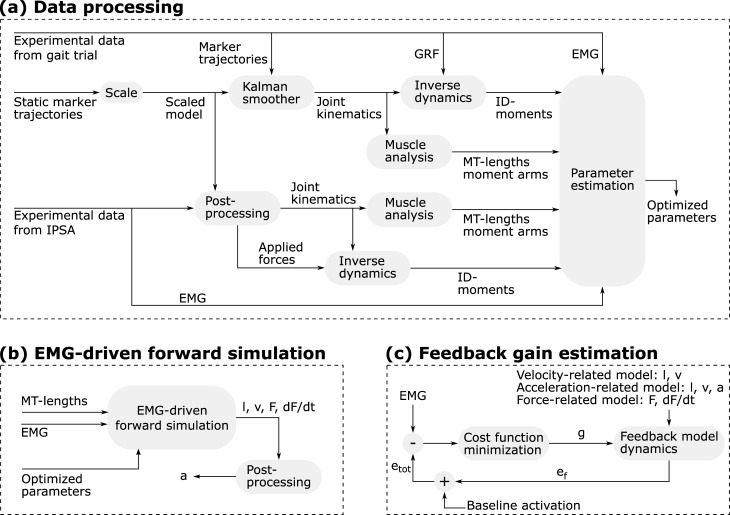
**Flowcharts illustrating data processing (a), EMG-driven forward simulation (b), and feedback gain estimation (c).** (a) Experimental data from gait trials and IPSA at slow velocities are processed using musculoskeletal modeling to obtain muscle-tendon (MT) lengths, moment arms, and inverse dynamic (ID) joint moments that are input to the EMG-driven muscle-tendon parameter estimation. (b) Sensory information (l: muscle fiber length, v: muscle fiber velocity, F: muscle force, dF/dt: first time derivative of muscle force, a: muscle fiber acceleration) is obtained through EMG-driven forward simulations using MT-lengths, EMG, and optimized muscle-tendon parameters as input. EMG-driven forward simulations are performed for passive stretches at medium and fast velocities and for walking trials. (c) Feedback gains g are estimated by minimizing the difference between EMG and simulated excitation e_tot_, which is the sum of baseline activation and muscle excitation resulting from proprioceptive feedback e_f_. The feedback gain estimation is performed using experimental data from three fast passive stretches.

In this section, we detail the data processing steps that preceded the development and evaluation of the spasticity models (Fig 1A).

We processed the experimental data from IPSA and gait trials in OpenSim 3.3 [29] using the gait2392 musculoskeletal model containing 20 segments, 23 DOFs, and 43 Hill-type muscle-tendon actuators per leg [30] (Fig 1A). The musculoskeletal model was scaled to the subjects’ anthropometry using OpenSim’s Scale tool based on marker information collected during a static trial performed during the gait analysis. The maximal isometric muscle forces were scaled to the subjects’ mass. For each gait trial, joint kinematics were calculated from the measured marker trajectories using a Kalman smoothing algorithm [31]. Inverse dynamic joint moments were calculated from joint kinematics and measured GRFs using OpenSim’s Inverse Dynamics tool. For each passive motion (IPSA), experimental joint angles and forces applied by the examiner, pre-processed using custom-made software [7], were imported in OpenSim and inverse dynamic joint moments were calculated using the Inverse Dynamics tool. Muscle-tendon lengths and moment arms were computed for joint kinematics during gait and passive motions using OpenSim’s Muscle Analysis tool.

We normalized EMG based on estimated muscle excitation. We computed muscle excitation that reproduced the inverse dynamic moments during gait by minimizing muscle excitation squared while accounting for Hill’s muscle-tendon dynamics (including a compliant tendon) [32]. For each gait trial and muscle, we determined the normalizing factor as the ratio between peak EMG and peak estimated muscle excitation. For the passive motions, we normalized the EMG envelopes using the individuals’ largest normalizing factor from the gait trials (within-subject differences between normalizing factors for different gait trials were small, within-subject standard deviation of normalizing factors averaged over all subjects: 0.05 ± 0.05).

To account for inter-subject differences in muscle-tendon properties, we estimated subject-specific tendon slack lengths and optimal muscle fiber lengths of eight knee muscles (four knee extensors: biceps femoris long head, gastrocnemius lateralis and medialis, semimembranosus and four knee extensors: rectus femoris, vastus intermedius, lateralis, and medialis) by optimizing the fit between inverse dynamic and simulated joint moments (Fig 1A). Joint moments were simulated using an EMG-driven approach [33]. We used experimental data from three gait trials and from slow passive motions to optimize the parameters using the parameter estimation approach described by Falisse et al. [33]. Passive motions at medium and fast velocities were not used for the parameter estimation since only few EMG channels were available during these trials. For slow passive motions, we could assume low activity for muscles without EMG channels. However, this assumption does not necessarily hold for faster speeds, which motivated the exclusion of trials at faster speeds from the parameter estimation.

### Models of spasticity

We developed three spasticity models based on proprioceptive feedback. The first model relied on feedback from muscle fiber length and velocity, and is referred to as velocity-related model. The second model relied on feedback from muscle fiber length, velocity, and acceleration, and is referred to as acceleration-related model. The third model relied on feedback from muscle force and *dF*/*dt*, and is referred to as force-related model. Each feedback component, *e*_*s*_, is described by first order dynamics to model the time delay, *τ* = 30 ms [14]. The feedback is characterized by a threshold, *T*_*s*_, and a gain factor, *g*_*s*_:

$$\tau \frac{de_{s}}{dt}=\left\{ \begin{array}{c} -e_{s}\;s\leq T_{s}\\ -e_{s}+g_{s}(s-T_{s})\;s>T_{s} \end{array}\right.$$ (1)

where *s* refers to the sensory information, i.e. muscle fiber length, velocity, acceleration, muscle force, or *dF*/*dt*. For numerical reasons, we implemented a continuously differentiable approximation of Eq (1) using a hyperbolic tangent function to smoothly transition between feedback contributions above and below the threshold. We determined the thresholds for muscle fiber length, velocity, and muscle force as the values 20 ms before the EMG onset that we identified according to the method of Staude and Wolf [34]. We manually corrected the EMG onsets that were misidentified, likely due to filtering. This delay of 20 ms differs from the time delay τ = 30 ms because we used a first order approximation in the formulation of the feedback dynamics (Eq 1). We used zero thresholds for acceleration and *dF*/*dt* feedback, i.e. there is only feedback when the signal is positive.

We estimated the sensory information, i.e. muscle fiber length, velocity, acceleration, muscle force, and *dF*/*dt*, that is input to the spasticity models during passive motions at medium and fast velocities (for both hamstrings and gastrocnemii) by performing EMG-driven forward simulations of Hill’s muscle-tendon model dynamics (Fig 1B). Muscle fiber length, velocity, muscle force, and *dF*/*dt* follow directly from forward integration of the muscle-tendon dynamics (Hill’s model with personalized parameters) using the muscle’s EMG and muscle-tendon length trajectories as input. We used semitendinosus EMG to drive biceps femoris long head, semimembranosus, and semitendinosus. Similarly, we used gastrocnemius lateralis EMG to drive both gastrocnemius lateralis and medialis. We estimated muscle fiber acceleration through a spline approximation of the muscle fiber velocity that was low pass filtered (second-order dual pass Butterworth filter with cut-off frequency of 10Hz).

For the three spasticity models, we optimized the feedback gains of the spastic muscles by minimizing the difference between experimental EMG and modeled muscle excitation, *e*_*tot*_, over three fast passive motions, later referred to as calibration motions (Fig 1C):

$$\min\sum_{k=1}^{3}\int_{t_{i}}^{t_{f}}{\left(e_{tot}(t)-EMG(t)\right)}^{2}dt$$ (2)

where *k* is calibration motion index, *t* is time, and *t*_*i*_ and *t*_*f*_ define the time interval. Only fast motions were used for calibrating the feedback gains since stretches at other velocities did not consistently provoke spastic responses. Note that we could not use gait trials to determine the feedback gains, since not all muscle activity during walking is due to sensory feedback. We assumed that muscle activity during passive motions consisted of a constant baseline activation and the different feedback components. The baseline activation was the minimal EMG value during the 0.5 s preceding stretch onset. The values of *t*_*i*_ and *t*_*f*_ were manually selected such that the optimization time window was centered around the increase in EMG corresponding to the spastic response (average optimization time window: 0.7 ± 0.2 s and 0.9 ± 0.5 s for the hamstrings and gastrocnemii, respectively).

The optimization problems were solved numerically via direct collocation using GPOPS-II optimal control software [35] on a mesh of 300 equally spaced intervals per second using third order Legendre-Gauss-Radau collocation. The interior point solver IPOPT [36] was used to solve the resulting nonlinear programming problems (NLP) using second-order derivative information. The automatic differentiation software ADiGator [37] was used to provide the derivatives required by the NLP solver.

We then used the spasticity models with optimized feedback gains to compute the muscle excitation of the spastic muscles during the entire duration of the passive motions at fast velocities, i.e. calibration motions, and medium velocities. The passive motions at medium velocities were used for validation only and will be referred to as validation motions. For the validation motions, we used, for each subject, as feedback thresholds the smallest thresholds from the three calibration motions (differences between thresholds for each subject were small, within-subject standard deviation of thresholds averaged over all subjects: 0.01 ± 0.01, 0.02 ± 0.03, and 0.02 ± 0.01 for the normalized muscle force, muscle fiber length, and muscle fiber velocity, respectively). To simulate the excitation of the spastic muscles, we performed forward integration of the feedback model dynamics, i.e. spasticity models (Eq 1), using the *ode45* solver in MATLAB (The Mathworks Inc., Natick, USA).

### Spastic contribution during gait

We then used the spasticity models with optimized feedback gains to simulate muscle excitation due to spindle reflexes during the gait trials. To this aim, we first estimated sensory information during gait trials for the spastic muscles by performing EMG-driven forward simulations of Hill’s muscle-tendon dynamics (Fig 1B). We then performed forward integration of the spasticity models (Eq 1) to simulate muscle excitation due to spindle reflexes. We used, for each subject, the smallest thresholds from the three calibration motions as feedback thresholds. Both steps were performed in a similar way as described above for the passive stretches.

Code developed to optimize the muscle-tendon parameters, implement the spasticity models, optimize the feedback gains, and simulate the spastic contributions during passive motions and gait trials are available at https://simtk.org/home/simcpspasticity.

### Outcome measures

We first compared the ability of the spasticity models to explain EMG during IPSAs. We then evaluated how muscle excitation predicted by the spasticity models during gait correlated with measured EMG. During gait, we expect the muscle excitation resulting from the spasticity models to lie within the EMG envelope when a spastic contribution is expected but not to fully explain the EMG, motivating the selection of a correlation analysis rather than an analysis of the quality of fit.

**Fig 2:**
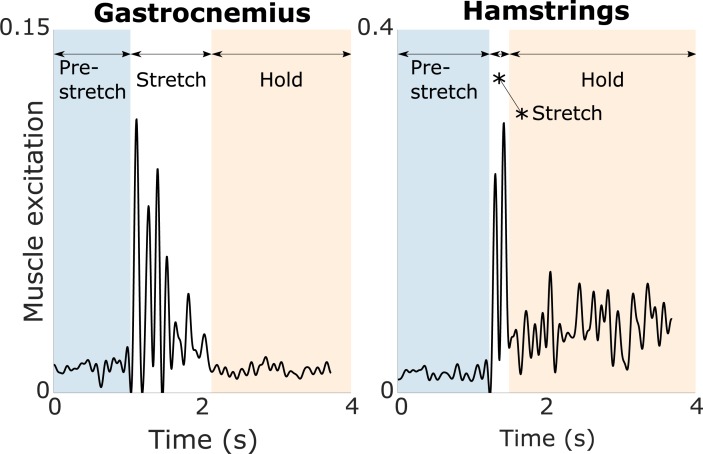
**Examples of measured muscle activity (EMG) during fast passive stretches of gastrocnemii (left) and hamstrings (right).** Typically, each stretch consists of a pre-stretch phase with low muscle activity corresponding to the baseline activation, a stretch phase with increased muscle activity corresponding to the spastic response, and a hold-phase with sustained muscle activity for the hamstrings and low muscle activity for the gastrocnemii.

For the IPSAs, we computed the root mean square error (RMSE) and the coefficient of determination (R^2^) between the EMG and the simulated muscle excitation over the entire duration of each calibration motion (average duration: 2.7 ± 0.7 s and 3.9 ± 1.0 s for the hamstrings and gastrocnemii, respectively) and validation motion (average duration: 2.7 ± 0.6 s and 2.6 ± 0.8 s for the hamstrings and gastrocnemii, respectively). Each motion included a pre-stretch phase, a stretch phase, and a hold phase (Fig 2). The duration of the pre-stretch and hold phases varied across trials, explaining why a passive motion at fast velocity may be longer than a passive motion at medium velocity. Typically, we observed low muscle activity, corresponding to the baseline activation, during the pre-stretch phase, an increased muscle activity, corresponding to the spastic response, during the stretch-phase, and a low or sustained muscle activity for the gastrocnemii or hamstrings, respectively, during the hold phase. We included all three phases when comparing the fits between models to evaluate the ability of the models to capture the entire muscle activity patterns. For the gait trials, we correlated the EMG and the simulated muscle excitation using a cross-correlation with zero-lag.

We then compared RMSE and R^2^ for IPSAs, and cross-correlations for gait trials between the three spasticity models using paired t-tests with a 0.05 significance level. We tested the IPSA movements used for calibration (fast velocity) and validation (medium velocity) separately. The samples included 8 and 12 values for the hamstrings and gastrocnemii, respectively.

## Results

**Fig 3:**
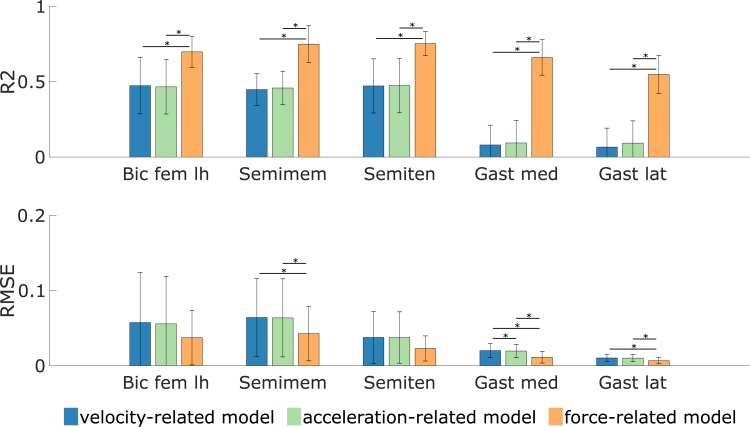
Fits (R^2^ and RMSE) between EMG and simulated muscle excitation during fast passive stretches. Muscle excitation is estimated based on the three spasticity models (velocity-, acceleration-, and force-related). A larger R^2^/smaller RMSE value represents a better fit. The statistical significance level was set to 0.05 and statistical differences between models are indicated using a horizontal bar. Results are averaged over 8 and 12 values for the hamstrings (biceps femoris long head (Bic fem lh), semimembranosus (Semimem), and semitendinosus (Semiten)) and gastrocnemii (gastrocnemius medialis (Gast med) and lateralis (Gast lat)), respectively.

The force-related spasticity model better explained the experimental EMG during the passive stretch motions at fast velocities than the velocity- and acceleration-related models (Fig 3). R^2^ values between EMG and simulated muscle excitation were higher using the force-related model (average 0.73 ± 0.10 and 0.60 ± 0.13 for the hamstrings and gastrocnemii, respectively) compared to the velocity-related model (average 0.46 ± 0.15 and 0.07 ± 0.13) and the acceleration-related model (average 0.47 ± 0.15 and 0.09 ± 0.14). RMSE were smaller using the force-related model (average 0.034 ± 0.031 and 0.009 ± 0.007 for the hamstrings and gastrocnemii, respectively) compared to the velocity-related model (average 0.053 ± 0.051 and 0.015 ± 0.009) and the acceleration-related model (average 0.052 ± 0.050 and 0.015 ± 0.008). The differences between the force-related model and the two other models were statistically significant (p<0.05) for all muscles in terms of R^2^ and for the semimembranosus, gastrocnemius medialis, and gastrocnemius lateralis in terms of RMSE. The differences were also statistically significant between the velocity- and acceleration-models for the gastrocnemius medialis in terms of RMSE. For the passive stretches at medium velocities, the force-related model also outperformed the two other models (S1 Fig). However, the fits between EMG and simulated muscle excitation were worse (smaller R^2^) than for the passive stretches at fast velocities.

**Fig 4:**
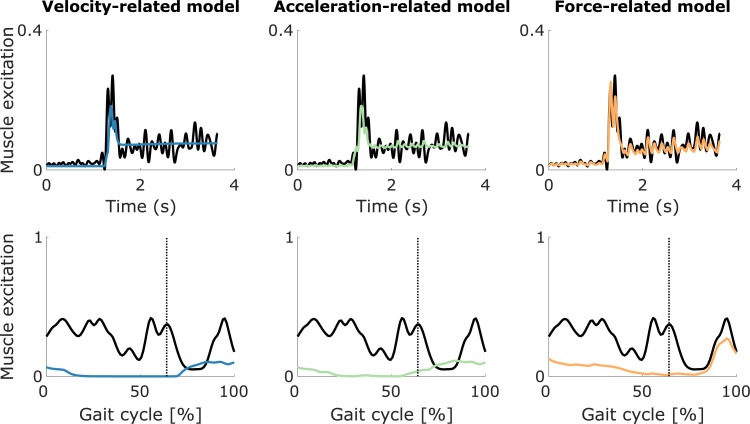
EMG compared to sensory feedback of the semimembranosus (hamstrings). The EMG (thick black lines) and sensory feedback (colored lines) are shown for one trial of a fast passive stretch motion (top) and a gait motion (bottom) for the semimembranosus (hamstrings) of one CP child. The velocity-related model combines muscle fiber length and velocity feedback. The acceleration-related model combines muscle fiber length, velocity, and acceleration feedback. The force-related model combines muscle force and dF/dt feedback. The three models also include a baseline activation during the fast passive stretch motion (top). The vertical lines indicate the transition from stance to swing (bottom). Other cases are shown in S2 Fig.

**Fig 5:**
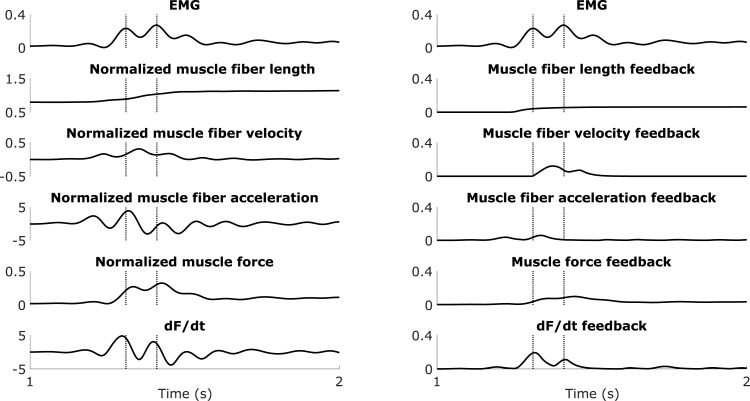
EMG, sensory information, and sensory feedback of the semimembranosus (hamstrings) during a fast passive stretch motion. The EMG (top), normalized sensory information (left), and corresponding sensory feedback (right) are shown for one trial of a fast passive stretch motion for the semimembranosus (hamstrings) of one CP child (corresponding to Fig 4 (zoom between 1 and 2 s)). The vertical lines indicate the first two EMG peaks. Normalized muscle fiber acceleration is the first time derivative of normalized muscle fiber velocity. dF/dt is the first time derivative of normalized muscle force.

Only the force-related model accurately described the oscillations in the measured EMG of both hamstrings and gastrocnemii. During fast passive stretches of the hamstrings, all three models could capture the salient features of the EMG (peak during stretch and sustained activity in hold phase) (example in Fig 4, top; other cases in S2 Fig) but only the force-related model could reproduce the distinct features of the signal (Fig 4, top-right). In all cases (example in Fig 5, right), we observed that delayed feedback from *dF*/*dt* explained the peaks in muscle activity during the stretch and the rapid changes (oscillations) in muscle activity throughout the response, whereas feedback from muscle force mainly contributed to the sustained baseline muscle activity after the stretch. Feedback from fiber velocity reproduced the increase in muscle activity upon stretch but the single peak of the simulated activity did not match the peaks in EMG. Feedback from fiber length contributed to the sustained baseline muscle activity after the stretch (Fig 4, top-left and top-middle, and S2 Fig). In three out of eight cases, feedback from fiber acceleration reproduced the rapid changes (oscillations) in muscle activity, although to a lesser extent than feedback from *dF*/*dt* (Fig 4, top-middle, and S2 Fig). In the other cases, estimated fiber acceleration feedback gains were very small.

**Fig 6:**
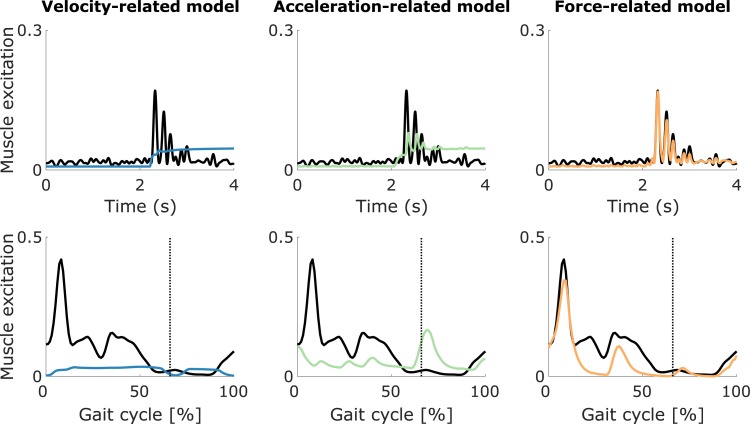
EMG compared to sensory feedback of the gastrocnemius medialis (gastrocnemii). The EMG (thick black lines) and sensory feedback (colored lines) are shown for one trial of a fast passive stretch motion (top) and a gait motion (bottom) for the gastrocnemius medialis (gastrocnemii) of one CP child. The velocity-related model combines muscle fiber length and velocity feedback. The acceleration-related model combines muscle fiber length, velocity, and acceleration feedback. The force-related model combines muscle force and dF/dt feedback. The three models also include a baseline activation during the fast passive stretch motion (top). The vertical lines indicate the transition from stance to swing (bottom). Other cases are shown in S3 and S4 Figs.

**Fig 7:**
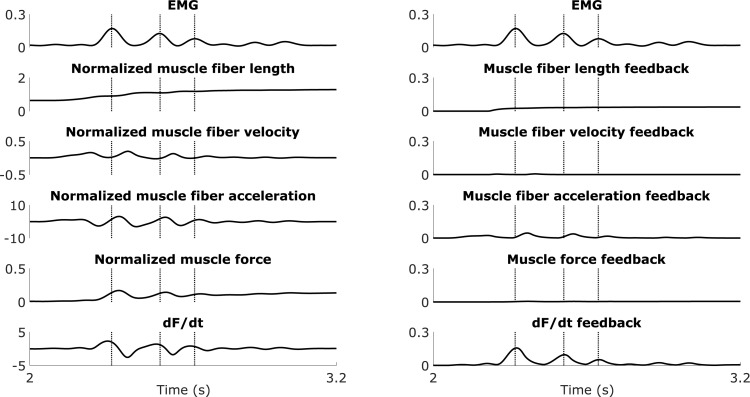
EMG, sensory information, and sensory feedback of the gastrocnemius medialis (gastrocnemii) during a fast passive stretch motion. The EMG (top), normalized sensory information (left), and corresponding sensory feedback (right) are shown for one trial of a fast passive stretch motion for the gastrocnemius medialis (gastrocnemii) of one CP child (corresponding to Fig 6 (zoom between 2 and 3.2 s)). The vertical lines indicate the first three EMG peaks. Normalized muscle fiber acceleration is the first time derivative of normalized muscle fiber velocity. dF/dt is the first time derivative of normalized muscle force.

During fast passive stretch motions of the gastrocnemii, the force-related model could reproduce the EMG whereas the two other models could not even capture the overall shape of the signal (example in Fig 6, top; other cases in S3 and S4 Figs). In all cases (example in Fig 7, right), delayed feedback from *dF*/*dt* reproduced the large peaks in muscle activity during the stretch and the smaller oscillations in muscle activity during the pre-stretch and hold phases, whereas muscle force feedback contributed to the low/sustained muscle activity after the stretch. In five out of twelve cases, fiber velocity feedback resulted in a single peak in muscle activity that was out of phase with the EMG and had a lower amplitude than the peak EMG. Estimated velocity feedback gains were very small in the other cases. In all cases, fiber length feedback mainly contributed to the low/sustained muscle activity after the stretch (Fig 6, top-left and top-middle, and S3 and S4 Figs). In six out of twelve cases, fiber acceleration feedback reproduced the oscillations in EMG during the stretch to some extent, although the amplitude of the simulated muscle activity was a lot smaller than the amplitude of the EMG, whereas estimated acceleration feedback gains were very small in the other cases (Fig 6, top-middle, and S3 and S4 Figs).

**Fig 8:**
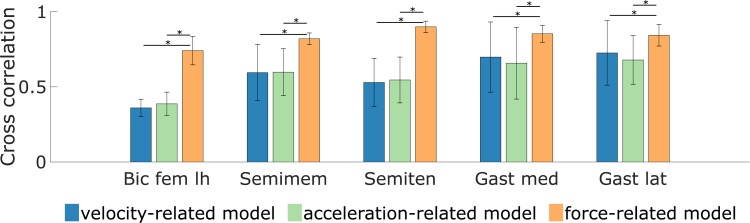
Cross-correlations between EMG and simulated muscle excitation during gait. Muscle excitation is estimated based on the three spasticity models (velocity-, acceleration-, and force-related). The statistical significance level was set to 0.05 and statistical differences between models are indicated using a horizontal bar. Results are averaged over 8 and 12 values for the hamstrings (biceps femoris long head (Bic fem lh), semimembranosus (Semimem), and semitendinosus (Semiten)) and gastrocnemii (gastrocnemius medialis (Gast med) and lateralis (Gast lat)), respectively.

Muscle activity predicted by the force-related spasticity model during gait was consistent with measured EMG whereas this was not the case for the velocity- and acceleration-related models (see Fig 4 and Fig 6 for examples; other cases in S2–S4 Figs). The agreement between predicted feedback muscle activity and measured EMG is reflected in the cross-correlations (Fig 8). The cross-correlations between EMG and predicted muscle activity were larger using the force-related model (average 0.82 ± 0.09 and 0.85 ± 0.06 for the hamstrings and gastrocnemii, respectively) compared to the velocity-related model (average 0.49 ± 0.17 and 0.71 ± 0.22) and the acceleration-related model (average 0.51 ± 0.16 and 0.67 ± 0.20). The differences between the force-related model and the two other models were statistically significant (p<0.05) for all muscles.

During gait, only the force-related spasticity model predicted muscle activity that lay within the EMG envelope for the hamstrings (example in Fig 4, bottom; other cases in S2 Fig). The force-related model predicted high muscle activity at the end of swing that resembled measured EMG and lower feedback muscle activity during stance that lay within the EMG envelope (in one out of eight cases, the predicted muscle activity exceeded the EMG at the end of swing) (Fig 4, bottom-right, and S2 Fig). In all cases, the velocity- and acceleration-related models predicted muscle activity during mid-swing that was not consistent with EMG (Fig 4, bottom-left and bottom-middle, and S2 Fig), i.e. the predicted muscle activity occurred when no EMG was measured. Sensory information and corresponding feedback contributions of the example in Fig 4 are in Supplementary Material (S5 Fig).

During gait, only the force-related model predicted activity of the gastrocnemii that lay within the EMG envelope and captured the abnormal peak in activity at initial contact that we observed in a subgroup of the subjects. A first group of subjects landed on their heel at initial contact (four out of twelve cases) whereas a second group of subjects landed on their forefoot instead of their heel at initial contact (eight out of twelve cases). In this second group, increased muscle activity, which is not observed in healthy individuals during walking, was measured at the beginning of stance (example in Fig 6, bottom; other cases in S4 Fig). In the first group, the force-related model predicted muscle activity during both stance and swing that lay within the EMG envelope whereas the two other models predicted low muscle activity (S3 Fig). In the second group, the force-related model predicted the abnormal increase in EMG during initial stance as well as muscle activity during mid-stance and at the end of swing that lay within the EMG envelope (Fig 6, bottom-right, and S4 Fig). The other two models predicted either low levels of muscle activity or muscle activity that lay outside the EMG envelope (Fig 6, bottom-left and bottom-middle, and S4 Fig). Sensory information and corresponding feedback contributions of the example in Fig 6 are in Supplementary Material (S6 Fig).

## Discussion

We showed that a spasticity model combining delayed feedback from muscle force and its first time derivative (*dF*/*dt*) (force-related model) could explain muscle activity measured during fast passive stretches of the hamstrings and gastrocnemii of children with spastic CP. Although spasticity has been defined as a velocity-dependent increase in stretch reflexes, our models combining feedback from muscle fiber length, velocity, and acceleration (velocity- and acceleration-related models) could not explain the measured muscle activity during passive stretches. In addition, we showed that the force-related model predicted muscle excitation during gait that was consistent with the muscle activity measured in the hamstrings and gastrocnemii of the CP children. Specifically, the force-related model predicted the abnormal peak in gastrocnemii activity in early stance that was observed in the subset of children that landed on their forefoot instead of on their heel. Our force-related model of spasticity may therefore be useful in quantifying gait impairments resulting from spasticity on a patient-specific basis.

Our results suggest that spastic muscle activity can be described by exaggerated feedback from muscle force and its first time derivative, and are therefore in agreement with a recent experimental study [15] that shows that muscle spindles encode muscle force and force rate. Spindle firing is traditionally thought to encode information about muscle fiber length and velocity. Fiber length and velocity are related to muscle force through the muscle’s force-length-velocity properties. During a stretch, the increased fiber velocity results in an increase in (eccentric) muscle force and, if the muscle is working on the ascending part of the force-length curve, the increased fiber length also results in an increase in muscle force. However, this relation is not linear and Blum et al. [15] demonstrated that spindle firing, measured *in vitro* during stretch of passive muscles, is uniquely related to muscle fiber force and its first time derivative but not to length and velocity. This observation is in line with older *in vitro* studies that related peaks in muscle spindle firing rates to muscle force transients at stretch onset [19,20]. Furthermore, our model is in agreement with existing physiology-inspired models of spindle firing that describe the sensory region of the spindle as a spring in series with the intrafusal muscle fibers, thereby modeling spindle firing as proportional to spring elongation, which is in turn proportional to muscle force [16–18]. Finally, our model is based on delayed sensory feedback and therefore the peak in *dF*/*dt* that mainly explains the peak in EMG (Figs 5 and 7) cannot solely be a response of altered muscle activity. Instead, the muscle’s state is altered by the imposed stretch and this is reflected in the EMG with a delay. Further delays are also introduced by muscle activation and contraction dynamics. However, there is a need for additional studies documenting force encoding in muscle spindles.

Our predictions of reflex muscle activity during gait based on feedback from force-related variables suggest that spasticity contributes to gait impairments in CP. Several studies argued that spasticity of the hamstrings might contribute to crouch gait, a typical pathological gait pattern in CP children [38], by restricting knee extension at terminal swing [25,38–41]. Our simulation results demonstrate that exaggerated proprioceptive feedback from muscle force and *dF*/*dt* may indeed contribute to hamstrings activity at the end of swing (see example simulation of a subject with crouch gait in Fig 4). Landing on the forefoot instead of the heel during initial contact is another common pathological deviation from normal walking seen in CP children. This gait deviation has been correlated to abnormally large stretch responses in the gastrocnemii [24]. In these cases, there is a large peak in gastrocnemius activity at about 8% of the gait cycle whereas the peak muscle activity in typical walking is expected at about 40% of the gait cycle [42]. Our results demonstrate that exaggerated feedback from *dF*/*dt* may cause the peak in gastrocnemius activity during early stance. Hence, modeling spasticity based on force-related variables allowed us to directly relate measures of spasticity in passive muscles to muscle activity during walking, providing further support for the role of spasticity in impeding gait performance in CP.

We could explain measured muscle activity during passive stretches and gait based on the same feedback model parameters (reflex thresholds and gains), which is in agreement with a lack of or decrease in reflex modulation reported for patients with spasticity [4,43–45]. In healthy individuals, reflex modulation has been shown to be context-dependent whereas it has been suggested that supraspinal input is dysregulated in patients with CP [44,46–49]. As examples, Faist et al. [44] reported less quadriceps reflex depression and modulation in patients with spasticity due to cerebral lesions during gait as compared to healthy subjects and Sinkjaer et al. [45] reported less soleus stretch reflex modulation during gait in patients with spasticity due to multiple sclerosis. Furthermore, Nielsen et al. [50] provided a reasoning that supports our approach of using the same spasticity model in passive motions and gait. On the one hand, stretch reflex activity is larger in active contractions than in passive conditions due to depression of the inhibitory mechanisms in healthy subjects in active conditions. On the other hand, inhibitory mechanisms are already depressed in passive conditions in spastic individuals. Thus, these mechanisms cannot be depressed further during active contractions in spastic individuals leading to similar reflexes during passive and active conditions.

It is likely that not all reflex muscle activity predicted by the spasticity model during gait is pathological. In particular, the hamstrings are active at the end of swing in normal walking to modulate the rate of knee extension and prepare the leg for stance [42] and this activity has been attributed to stretch reflexes [51]. It is therefore hard to dissociate normal and pathological muscle activity. It is possible that the feedback pathways used to model spasticity in this study are part of the neural circuitry responsible for normal gait that is inhibited in passive conditions and modulated throughout the gait cycle in typically developed individuals. However, our spasticity model that was calibrated based on muscle activity during passive motions that has been attributed to spasticity, also predicted muscle activity during gait that matched experimental EMG of CP children but is not observed in healthy individuals, e.g. burst in gastrocnemius activity at the beginning of stance.

Our spasticity models do not differentiate between hypersensitivity of the muscle spindles, i.e. increased muscle spindle firing rates, and hyperexcitability of the motoneuron pool. Instead, muscle spindle firing and motoneuron excitability are lumped in our simple models that describe muscle spindle reflexes by delayed sensory feedback. Our results suggest that force encoding in muscle spindles underlies spastic muscle activity but should not be interpreted as spasticity being primarily due to changes in muscle spindle sensitivity rather than spinal cord and supraspinal dysregulation. Previous studies that separately modeled the muscle spindle and neuromotor pool suggested that alterations in motoneuron excitability and not muscle spindle hypersensitivity is the primary cause of spasticity [52,53].

Fits between EMG and simulated muscle excitation were relatively low for the gastrocnemii for the passive motions at medium velocity that were not used for calibration, although the force-related model still outperformed the other models (average R^2^ and RMSE: 0.24 ± 0.21 and 0.014 ± 0.013, respectively). This is partly explained by the fact that, in several cases, the clinical tests did not reveal any spastic involvement at medium velocities, i.e. no EMG onsets were identified, which was poorly reproduced by the models and explains the large variability in the results. In the future, we might therefore need to refine our model and our approach to determine the feedback thresholds. Other methods have been proposed in the literature. For example, instead of modeling reflex thresholds for each feedback component, Safavynia and Ting [54] reconstructed EMG signals based on linear feedback of the state variables by adding all feedback components and then half-wave rectifying the total signal. Such an approach might result in better fits by including information from negative signals. Overall, also for the stretches at medium velocity the force-related model outperformed the other models and captured the distinct features of the spastic response in most cases.

We made several assumptions when developing the spasticity models. First, we used muscle-tendon force feedback whereas spindles presumably sense active muscle force [15]. Muscle-tendon force is nevertheless a good surrogate for active muscle force if passive force and pennation angle are small, as in our case. Yet, future work should consider using active muscle force rather than muscle-tendon force. Second, we used a fixed delay of 30 ms for all feedback pathways. Using larger delays could have increased the quality of the fit between EMG and muscle excitation resulting from the velocity feedback for the gastrocnemii. However, having the peak velocity feedback matching the peak EMG would have required delays exceeding reported values [55]. Third, there was no attempt to model the influence of gamma drive on the spindle sensitivity. Nielsen et al. [50] reported that the increased gamma-motor activity should not play a role in spasticity. Fourth, we did not explicitly constrain feedback muscle activity to muscle lengthening, which could be a valid assumption since spasticity models aim to simulate exaggerated muscle activity resulting from hyper-excited stretch reflexes. However, we verified that this would have had little influence on the main conclusions. In the example of Fig 4, the only noticeable difference would have been an inhibition of the feedback contributions observed in the stance phase for the hamstrings since the latter are shortening during that phase (S5 Fig). The influence on the gastrocnemii would have been minor. Constraining feedback muscle activity to phases where the muscle is lengthening would therefore not change our conclusions. Fifth, the EMG processing workflow might influence the results. However, we verified that increasing the low-pass cut-off frequency had little influence on the main conclusions. Finally, we did not model the increased muscle stiffness that is known to contribute to muscle hyper-resistance in CP children [14]. However, we estimated subject-specific muscle-tendon parameters to reproduce inverse dynamic moments during slow passive stretches and gait. Since inverse dynamic moments and moments simulated using the optimized parameters were in good agreement for the slow passive stretches, there was no need to further model the increased passive stiffness.

## Conclusion

In this study, we proposed a spasticity model based on delayed feedback from muscle force and its first time derivative that could explain muscle activity from instrumented passive spasticity assessments. Additionally, the model could predict hamstrings and gastrocnemii activity during gait in spastic CP children that was consistent with measured muscle activity. Using personalized models of spasticity may be important to advance our understanding of movement impairments due to spasticity. Moreover, inclusion of spasticity models in predictive simulations of walking may result in more accurate simulations of walking kinematics that can eventually be used to predict treatment outcome in children with CP.

## Supporting information

S1 Fig Fits (R2 and RMSE) between EMG and simulated muscle excitation during passive stretches at medium velocity. Muscle excitation is estimated based on the three spasticity models (velocity-, acceleration-, and force-related). A larger R^2^/smaller RMSE value represents a better fit. The statistical significance level was set to 0.05. Results are averaged over 8 and 12 values for the hamstrings (biceps femoris long head (Bic fem lh), semimembranosus (Semimem), and semitendinosus (Semiten)) and gastrocnemii (gastrocnemius medialis (Gast med) and lateralis (Gast lat)), respectively.
(EPS)

S2 Fig EMG compared to sensory feedback of the semimembranosus (hamstrings). The EMG (thick black lines) and sensory feedback (colored lines) are shown for one trial of a fast passive stretch motion (top) and a gait motion (bottom). The velocity-related model combines muscle fiber length and velocity feedback. The acceleration-related model combines muscle fiber length, velocity, and acceleration feedback. The force-related model combines muscle force and dF/dt feedback. The three models also include a baseline activation during the fast passive stretch motion (top). The vertical lines indicate the transition from stance to swing. Each box (a-g) represents a different case.
(EPS)

S3 Fig EMG compared to sensory feedback of the gastrocnemius medialis (gastrocnemii). The EMG (thick black lines) and sensory feedback (colored lines) are shown for one trial of a fast passive stretch motion (top) and a gait motion (bottom). The velocity-related model combines muscle fiber length and velocity feedback. The acceleration-related model combines muscle fiber length, velocity, and acceleration feedback. The force-related model combines muscle force and dF/dt feedback. The three models also include a baseline activation during the fast passive stretch motion (top). The vertical lines indicate the transition from stance to swing. Each box (a-d) represents a different case. In all cases, the CP children landed on their heel.
(EPS)

S4 Fig EMG compared to sensory feedback of the gastrocnemius medialis (gastrocnemii). The EMG (thick black lines) and sensory feedback (colored lines) are shown for one trial of a fast passive stretch motion (top) and a gait motion (bottom). The velocity-related model combines muscle fiber length and velocity feedback. The acceleration-related model combines muscle fiber length, velocity, and acceleration feedback. The force-related model combines muscle force and dF/dt feedback. The three models also include a baseline activation during the fast passive stretch motion (top). The vertical lines indicate the transition from stance to swing. Each box (a-g) represents a different case. In all cases, the CP children landed on their forefoot and we therefore observe a large abnormal peak in activity at initial contact.
(EPS)

S5 Fig EMG, sensory information, and sensory feedback of the semimembranosus (hamstrings) during a gait trial. The EMG (top), normalized sensory information (left), and corresponding sensory feedback (right) are shown for one gait trial for the semimembranosus (hamstrings) of one CP child (corresponding to Fig 4). The vertical lines indicate the transition from stance to swing. Normalized muscle fiber acceleration is the first time derivative of normalized muscle fiber velocity. dF/dt is the first time derivative of normalized muscle force.
(EPS)

S6 Fig EMG, sensory information, and sensory feedback of the gastrocnemius medialis (gastrocnemii) during a gait trial. The EMG (top), normalized sensory information (left), and corresponding sensory feedback (right) are shown for one gait trial for the gastrocnemius medialis (gastrocnemii) of one CP child (corresponding to Fig 6). The vertical lines indicate the transition from stance to swing. Normalized muscle fiber acceleration is the first time derivative of normalized muscle fiber velocity. dF/dt is the first time derivative of normalized muscle force.
(EPS)
